# Benchmarking pathway interaction network for colorectal cancer to identify dysregulated pathways

**DOI:** 10.1590/1414-431X20175981

**Published:** 2017-03-30

**Authors:** Q. Wang, C.-J. Shi, S.-H. Lv

**Affiliations:** 1 Department of General Surgery, Shanxi Provincial People's Hospital, Taiyuan, Shanxi Province, China; 2 Department of Endocrinology, The Second Affiliated Hospital of Mudanjiang Medical University, Mudanjiang, Heilongjiang Province, China; 3 Department of Gastroenterology, The Second Affiliated Hospital of Mudanjiang Medical University, Mudanjiang, Heilongjiang Province, China

**Keywords:** Pathway interaction network, Colorectal cancer, Dysregulated pathways, Principal component analysis, Protein-protein interaction

## Abstract

Different pathways act synergistically to participate in many biological processes. Thus, the purpose of our study was to extract dysregulated pathways to investigate the pathogenesis of colorectal cancer (CRC) based on the functional dependency among pathways. Protein-protein interaction (PPI) information and pathway data were retrieved from STRING and Reactome databases, respectively. After genes were aligned to the pathways, each pathway activity was calculated using the principal component analysis (PCA) method, and the seed pathway was discovered. Subsequently, we constructed the pathway interaction network (PIN), where each node represented a biological pathway based on gene expression profile, PPI data, as well as pathways. Dysregulated pathways were then selected from the PIN according to classification performance and seed pathway. A PIN including 11,960 interactions was constructed to identify dysregulated pathways. Interestingly, the interaction of mRNA splicing and mRNA splicing-major pathway had the highest score of 719.8167. Maximum change of the activity score between CRC and normal samples appeared in the pathway of DNA replication, which was selected as the seed pathway. Starting with this seed pathway, a pathway set containing 30 dysregulated pathways was obtained with an area under the curve score of 0.8598. The pathway of mRNA splicing, mRNA splicing-major pathway, and RNA polymerase I had the maximum genes of 107. Moreover, we found that these 30 pathways had crosstalks with each other. The results suggest that these dysregulated pathways might be used as biomarkers to diagnose CRC.

## Introduction

Colorectal cancer (CRC) affects millions of people in the world, and is the second most common cause of cancer-induced deaths in males and the third in females (1). It is characterized by the accumulation of epigenetic and genetic events, and is affected by lifestyle factors (2,3). Despite the existence of screening and preventive strategies, CRC remains a major public health problem. Of note, approximately 102,480 people were affected by and 50,830 died of CRC in the United States in 2013 (4). Death from CRC can be prevented by early stage detection, but it is often found at an advanced stage (5). Thus, understanding the pathogenic processes of CRC is essential for its early detection and treatment.

Recently, high throughput microarray technology exerted significant advances in the understanding of pathological mechanism of various diseases. In recent years, studies have created many microarray data associated with CRC; for example, the GSE4183 pathway, published by Galamb and colleagues (6), who evaluated the gene expression, detected several important genes. Another former study also used this microarray data to identify significant pathways using a subpathway-based method (7). However, functional dependency among pathways was ignored.

As reported, signaling pathways rather than individual genes govern the process of tumorigenesis and progression (8). With the goal of identifying signatures for early detection, many studies have examined the relationship of signaling pathways and CRC progression. For example, CRC has been indicated to be mainly associated with chromosome instability (9) and microsatellite instability pathways (10,11). However, there is a lack of understanding of mechanisms underlying the progression of CRC. More than one pathway might participate in a disease because of the complicated feature of biological systems. Two or more pathways may have crosstalks to induce disease, since functional proteins may participate in multiple pathways (12). Thus, in addition to extracting concrete pathways, detecting crosstalk between pathways that are associated with CRC might be more efficient. Of note, network-based methods are broadly employed to analyze interactions, thereby further shedding light on molecular mechanisms (13,14). Moreover, protein-protein interactions (PPIs) are used to establish a global interaction network that exhaustively describes the overall relationships among functionalities. Hence, we combined pathway data and PPI network to build a pathway interaction network (PIN) and further identify dysregulated pathways, a method which considers the functional dependency between pathways (15). Collectively, the dysregulated pathways will provide insight into the pathogenetic mechanisms of CRC and provide clues for disease therapy (16,17).

## Material and Methods

### Datasets

*Microarray data.* Gene expression profile for CRC with the accession NO. GSE4183 were retrieved from GEO database (https://www.ncbi.nlm.nih.gov/geo/) (6). This dataset compared various colorectal diseases (15 CRC samples, 15 inflammatory bowel diseases, 15 colon adenoma) with normal controls (n=8). In the current study, to explore the molecular mechanisms of CRC progression, we only selected 8 normal controls and 15 CRC samples for subsequent analysis. After the probes were mapped to the gene symbols, a total of 20,545 genes were identified. Then, the standardization of expression levels in all genes was implemented based on the equation:

$$Z_{mn}=\frac{g_{mn}-ave\left(g_{m}\right)}{std\left(g_{m}\right)}$$

In this equation, *gmn* stands for the expression level of gene *m* in sample *n*, ave(gm) and std(gm) are the average and standard deviation (SD) of the expression value of gene *m* in all samples, respectively.

*PPI network and pathway data.* All PPI information, about 16,730 proteins and 787,896 interactions, was retrieved from the STRING database (http://string-db.org) (18). STRING database applies confidence scores to estimate the probability that an association really exists. Thus, in an attempt to minimize the ambiguity, we only selected the interactions with confidence scores greater than 0.2 to establish the background PPI network. Next, the intersection of the background PPI network and microarray data were used to construct a targeted PPI network for following analysis.

At the same time, all human pathways (1,675 pathways) were extracted from Reactome database (http://www.reactome.org), which is an online curated resource for human pathway data (19). As pathways with too few genes might not have sufficient biological information, we generated a set of pathways by discarding the ones with less than 5 genes. Overall, we ended up with 1189 informative pathways.

### Pathway activity and PIN construction

This method identified dysregulated pathways via three steps. We first computed the activity of each pathway based on gene expression information, and selected a seed pathway. Then, we built a PIN relying on biological pathways and PPIs. Finally, the dysregulated pathways were extracted from the PIN according to classification performance and the seed pathway. The specific conditions were listed as follows.

*Calculation of pathway activity and selection of seed pathway.* In our study, we only reserved genes that were mapped to the 1189 informative pathways for further analysis. After the genes were aligned to the informative pathways, we determined an activity score for each pathway as the sum of the expression levels of all genes enriched in this given pathway. Principal component analysis (PCA) (20) was employed to obtain the summary of expression scores of all genes of each pathway. The activity score of pathway *k* in sample *n* was determined by the following formula:

$$P_{kn}=w_{1nk}z_{1nk}+w_{2nk}z_{2nk}\ldots +w_{mnk}z_{mnk}$$

in which, *wmnk* is the weight for *zmnk*, and *zmnk* and denotes the standardized expression level of gene *m* from pathway *k* in sample *n*. The first principal component obtained from PCA was defined as the activity score for the appropriate pathway. Thus, the pathways with different activity scores in disease and normal samples were possibly connected with disease progression. Thus, if the activity score for a defined pathway is different between the CRC and control samples, it indicates the relationship of CRC with this pathway. The greater this difference, the more relevant the pathway is to CRC progression. In the current study, the pathway with the maximum change in activity score between disease and control groups was defined as the seed pathway.

*Construction of PIN.* In order to determine whether a gene was expressed in a differential way, Student's *t*-test was employed to compare gene expression between the two conditions. We considered gene expressions significantly different between disease and control conditions if the P value was less than 0.05. Moreover, we calculated the Pearson correlation coefficient (PCC) for all PPI interactions between the two conditions, thereby having a distribution of the PCC. We also computed the difference in absolute values of the PCC for the PPI interactions in CRC and normal groups.

Based on microarray profile, pathway information and PPI data, we then established a PIN, where each node denoted a pathway, and an edge was connected between pathways if they met one of the two criteria. Otherwise, the edges would be abandoned. One criterion was that two pathways shared one or more common genes, and one or more of these genes between pathways were differentially expressed in CRC and control groups. The other criterion was that two genes that coded interacting proteins employed to connect an edge between pathways were highly co-expressed (PCC, absolute value greater than 0.8). Otherwise, the edges would be discarded. If a network is too large, a certain number of significant genes and interactions can be neglected (21). Thus, with the goal of reducing the intricate network, the score values of each pathway pair in the PIN were calculated, defined as the summation of the PCC absolute values for the PPIs in every two pathways. Then, we selected the top 5% pathway interactions to construct a targeted PIN for identifying dysregulated pathways.

*Identifying dysregulated pathways from the targeted PIN.* After computing the activity score for every pathway, we extracted the dysregulated pathways using a machine-learning framework, in which the minimum pathways that could best distinguish between diseases and controls were regarded to be potentially dysregulated pathways. For this selection process, support vector machines (SVMs) were utilized. An individual pathway, which best distinguished between disease and control, was first selected as seed pathway. Then, other pathways that had crosstalks with the seed pathway were combined with the seed pathway to obtain better classification accuracy. New pathways were selected when no pathways could be combined for better classification results. The ultimate extracted pathway set was kept as possibly dysregulated pathways of disease. We adopted the area under the curve (AUC) score as a measure of classification performance, and used five-fold cross validation to test the performance ability. In the cross validation, four of the five samples were employed as training set and the other was used as test set to assess the classification ability. With the goal of getting robust results, five-fold cross-validation was repeated 100 times, and then the mean level was used as the eventual result.

## Results

### Constructing the targeted PIN

**Figure 1:**
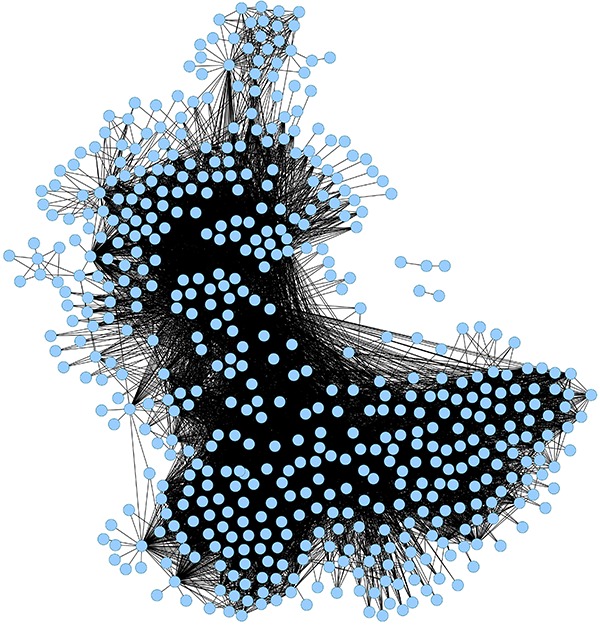
Pathway interaction network for colorectal cancer samples. Nodes represent pathways, and edges represent the interaction between any two pathways.

**Figure 2:**
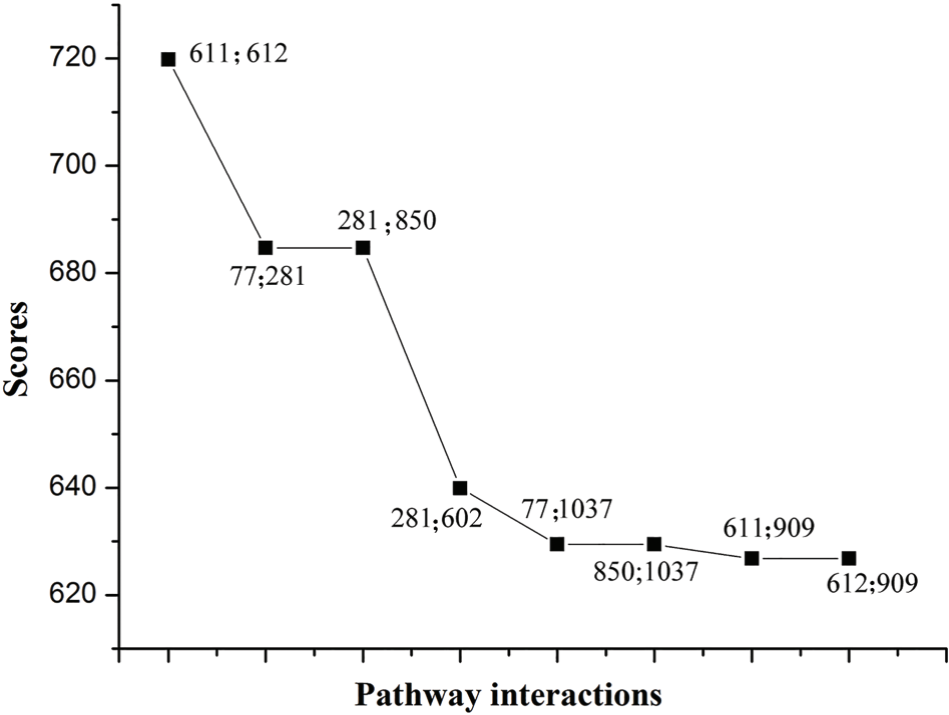
Score distribution of the top 8 pathway interactions with scores higher than 600; 77, APC/C-mediated degradation of cell cycle proteins; 281, DNA replication; 602, mitotic prometaphase; 611, mRNA splicing; 612, mRNA splicing-major pathway; 850, regulation of mitotic cell cycle; 909, RNA polymerase II transcription; 1037, synthesis of DNA. The numbers represent the pathway IDs, which were defined based on alphabetical order.

With the P values set at 0.05, we obtained 6,201 differentially expressed genes (DEGs) between CRC and control groups. The top 20 DEGs are shown in Table 1. These DEGs were applied to extract the interactions for constructing the PINs, since only interactions in the background PPI network met at least one of the two criteria and were kept to establish the PIN. After selecting edges, an original PIN was constructed, which covered 239,216 interactions among pathways. Since an intricate network easily ignores a few significant interactions (22), the complicated network should be reduced. In our analysis, the interactions with low |PCC| scores were discarded, and only the top 5% pathway interactions were extracted to construct a targeted PIN for selecting dysregulated pathways. In our analysis, as shown in Figure 1, the targeted PIN included 11,960 interactions among 1189 informative pathways. From this figure, we found that pathways interacted with each other, but the weight values were different. The weight value for a pathway-pathway interaction was defined as the total |PCC| scores of all genes, and interactions having higher weight values might be more important than the others for CRC. The weight scores among 11,960 interactions ranged from 95 to 720. Interestingly, we observed that 8 pathway interactions had the weight values higher than 600, as shown in Figure 2. Among these 8 pathway interactions, the DNA replication pathway (ID: 281) interacted with three pathways, i) APC/C-mediated degradation of cell cycle proteins (ID: 77), ii) regulation of mitotic cell cycle (ID: 850), and iii) mitotic prometaphase (ID: 602). Moreover, the interaction of mRNA splicing (ID: 611) and mRNA splicing-major pathways (ID: 612) had the highest scores with weight value of 719.8167 among the 8 pathway interactions.

**Table 1:**
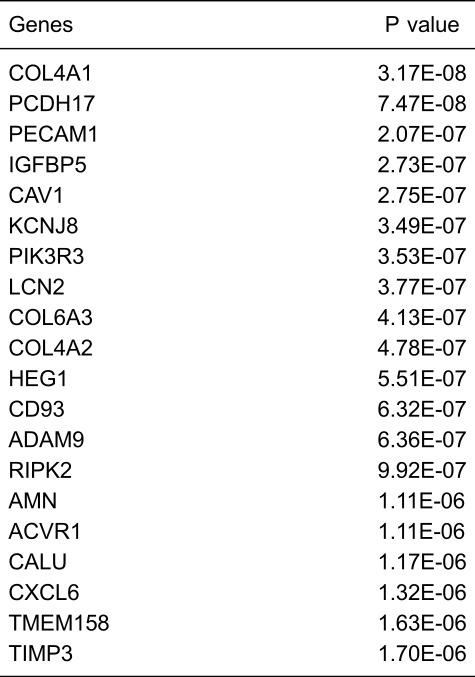
List of the top 20 differentially expressed genes (DEGs).

### Identifying dysregulated pathways

In this analysis, with the goal of evaluating the significance of pathways, the activity score for the 1189 pathways was calculated using the PCA method. The pathway with the maximum change of activity score between CRC and normal samples was defined as seed pathway. Of note, we found that the DNA replication pathway (ID: 281; statistic value=28.0527) showed the maximum change in the activity score between CRC and normal groups, and was selected as the seed pathway. Beginning with this seed pathway, the selection of dysregulated pathways was implemented according to the classification accuracy. This procedure stopped when classification accuracy did not increase. In our study, with "DNA replication" as initial, one pathway set containing 30 dysregulated pathways was obtained with AUC score of 0.8598, which indicated that these selected dysregulated pathways can be utilized as robust bio-signatures. The specific data is shown in Table 2. The mRNA splicing pathway (ID: 611), mRNA splicing-major pathway (ID: 612), and RNA polymerase I (ID: 905) owned the maximum of 107 genes. DNA replication (ID: 281) had a maximum number of 102 genes.

**Table 2:**
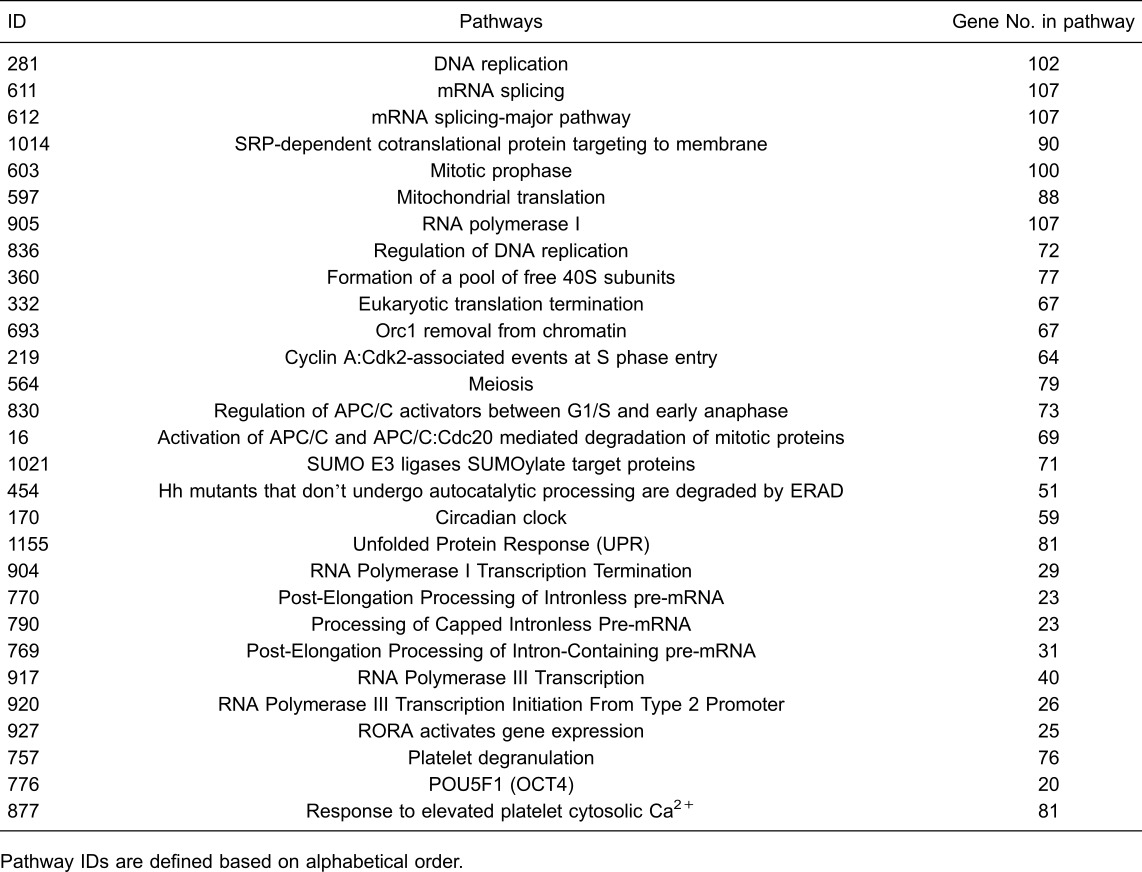
Pathway interaction network for colorectal cancer samples. Nodes represent pathways, and edges represent the interaction between any two pathways.

**Figure 3:**
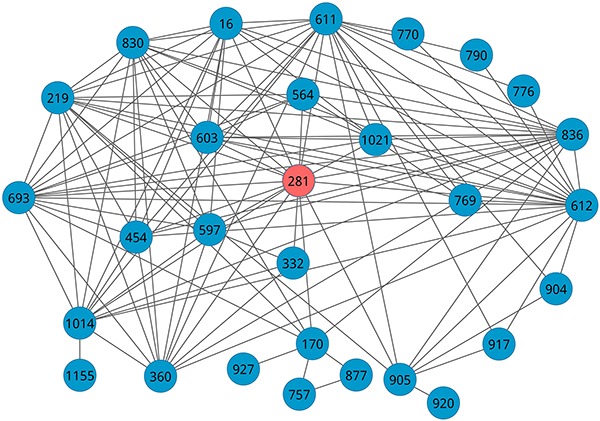
Dysregulated pathway interaction network in colorectal cancer. The 30 dysregulated pathways were assembled into a network according to the interactions. Each node represents a pathway. The orange node represents the seed pathway. Blue nodes are the dysregulated pathways interacted with the seed pathway. The numbers represent the pathway IDs defined based on alphabetical order.

In order to better present the relationship among these 30 identified dysregulated pathways in the PIN, we further explored their relationship and found that these pathways were assembled into a PIN network based on interactions. Figure 3 exhibits these interactions and their crosstalks.

## Discussion

Currently, pathway analysis has become the first choice to elaborate the biological functions of genes, since it increases explanatory power (22). Traditionally, pathway analysis mainly paid attention to single dysregulated pathways, and interactions among pathways were not considered (23). Broadly speaking, different pathways act synergistically to participate in many biological processes. Detecting pathway crosstalk is beneficial for studying pathway functions (24). Moreover, network biology offers new chances to analyze the interaction data and shed light into mechanisms by which cellular systems operate (25). Analyzing disease-associated interaction networks will be helpful for understanding the complicated cellular pathways and reveal disease progression processes. As a consequence, PIN was used in our study to extract dysregulated pathways relying on the pathway crosstalks via integrating PPI data and pathway information. One merit of this method is that when pathways have marginal P values, they still might carry a strong signal if they can develop a cluster in the PIN.

CRC is the third main cause of cancer-related-deaths in developed countries. Thus, it is understandable that major efforts have been done for dissecting the potential mechanisms underlying this disease. In our study, in order to expound the molecular mechanisms of CRC, we extracted the dysregulated pathways able to distinguish CRC from normal control samples, based on the constructed PIN that presented the functional dependency among pathways. We observed 1 pathway set with AUC of 0.8598 between CRC and normal samples, which demonstrated a good performance and the ability of this method to select dysregulated pathways in CRC. Of note, in this pathway set, there were 30 dysregulated pathways, such as mRNA splicing, mRNA splicing-major, RNA polymerase I, and DNA replication pathways, that owned a high number of pathway genes, and these pathways had crosstalks with each other. These results suggest that these dysregulated pathways might be useful as biomarkers to diagnose CRC.

In our study, maximum change of the activity score between CRC and normal groups occurred in the pathway of DNA replication, which was selected as the seed pathway. In the eukaryotic cell, DNA is replicated only once every cell cycle. The regulation of DNA replication is very important since loss of DNA replication control could threaten genome stability (26), a feature in most human cancers (27,28). In addition, chromosomal instability, as one form of genomic instability, is induced by defects in chromosomal segregation, DNA damage response, as well as telomere instability, which is a prominent pathway in CRC development and progression (9). Moreover, Pillaire and colleagues have indicated that DNA replication might be a new prognostic marker in CRC (29). Approximately 90% of genes in humans have been demonstrated to undergo alternative pre-mRNA splicing (30,31), which has been suggested to play important roles in a large number of human diseases (32). Spliceosome assembly appears co-transcriptionally, increasing the probability that DNA structure might affect alternative splicing (33). More crucially, it is evident that mRNA splicing is needed to promote transcript maturation and stability (34). In addition, the regulation of splicing has been indicated to be important in the response to DNA damage (34). DNA damage results in further genomic instability. Hence, the crosstalk between DNA replication and mRNA splicing might offer molecular basis for the occurrence and progression of CRC.

RNA polymerase I pathway is denoted uniquely to transcribe the copies of genes coding the pre-rRNA precursor, which is processed into 5.8S, 18S, and 28S rRNAs. Williamson et al. (35) reported that over-expression of rRNAs and pre-rRNAs is a characteristic of cancer. Bernstein et al. (36) suggested that there is a link between rRNA and cell proliferation. Of note, cancer is generally characterized by uncontrolled cell proliferation (37). Moreover, a former study has demonstrated that selective suppression of rRNA transcription hinders growth and proliferation of cancer cells (38). As demonstrated here, we infer that RNA polymerase I might be a useful target for CRC therapeutic strategy.

In conclusion, this integration-based analysis has several advantages. Unlike previous publications, we focused on the functional dependency between pathways by constructing a PIN, thereby indicating the robustness of our extracted pathway bio-signatures. Our findings suggest that dysregulated pathways, especially DNA replication and mRNA splicing, are important in CRC initiation, development and progression. However, several limitations must be taken into consideration. First, our sample size was very small. Second, the current study was analyzed based on existing data through bioinformatics methods, and the findings lacked experimental verifications. Thus, further investigations are warranted to verify the alterations of these pathways in animal experiments or patient tissues. Lastly, comparison between this novel method and other methods on multiple cancer datasets was not implemented to further demonstrate the effectiveness of our method. Thus, we expect our research to provoke further investigation into the potential roles of these dysregulated pathways in CRC. Despite these limitations, our results provided some preliminary evidence to uncover alterative candidate therapeutic strategies for CRC.

## References

[1] Jemal A, Bray F, Center MM, Ferlay J, Ward E, Forman D (2011). Global cancer statistics. CA Cancer J Clin.

[2] Giovannucci E (2003). Diet, body weight, and colorectal cancer: a summary of the epidemiologic evidence. J Womens Health.

[3] Migliore L, Migheli F (2011). Genetics, cytogenetics, and epigenetics of colorectal cancer. J Biomed Biotechnol.

[4] Tarver T (2012). American Cancer Society. Cancer facts and figures 2014. J Consumer Health Internet.

[5] Walsh JM, Terdiman JP (2003). Colorectal cancer screening: scientific review. JAMA.

[6] Galamb O, Györffy B, Sipos F, Spisák S, Németh AM, Miheller P (2008). Inflammation, adenoma and cancer: objective classification of colon biopsy specimens with gene expression signature. Dis Markers.

[7] Nam S, Park T (2012). Pathway-based evaluation in early onset colorectal cancer suggests focal adhesion and immunosuppression along with epithelial-mesenchymal transition. Plos One.

[8] Vogelstein B, Kinzler KW (2004). Cancer genes and the pathways they control. Nat Med.

[9] Pino MS, Chung DC (2010). The chromosomal instability pathway in colon cancer. Gastroenterology.

[10] Boland CR, Goel A (2010). Microsatellite instability in colorectal cancer. Clin Adv Hematol Oncol.

[11] Sinicrope FA, Sargent DJ (2012). Molecular pathways: microsatellite instability in colorectal cancer: prognostic, predictive, and therapeutic implications. Clin Cancer Res.

[12] Li Y, Agarwal P, Rajagopalan D (2008). A global pathway crosstalk network. Bioinformatics.

[13] Xia Y, Yu H, Jansen R, Seringhaus M, Baxter S, Greenbaum D (2004). Analyzing cellular biochemistry in terms of molecular networks. Ann Rev Biochem.

[14] Barabasi A-L, Oltvai ZN (2004). Network biology: understanding the cell's functional organization. Nat Rev Genet.

[15] Liu KQ, Liu ZP, Hao JK, Chen L, Zhao XM (2012). Identifying dysregulated pathways in cancers from pathway interaction networks. BMC Bioinformatics.

[16] Doniger S, Salomonis N, Dahlquist K, Vranizan K, Lawlor S, Conklin B (2003). MAPP finder: using gene ontology and GenMAPP to create a global gene-expression profile from microarray data. Genome Biol.

[17] Dawson JA, Kendziorski C (2012). An Empirical bayesian approach for identifying differential coexpression in high-throughput experiments. Biometrics.

[18] Szklarczyk D, Franceschini A, Kuhn M, Simonovic M, Roth A, Minguez P (2011). The STRING database in 2011: functional interaction networks of proteins, globally integrated and scored. Nucleic Acids Res.

[19] Croft D, O'Kelly G, Wu G, Haw R, Gillespie M, Matthews L (2011). Reactome: a database of reactions, pathways and biological processes. Nucleic Acids Res.

[20] Hotelling H (2010). Analysis of a complex of statistical variables into principal components. J Educ Psychol.

[21] Nibbe RK, Chowdhury SA, Koyutürk M, Ewing R, Chance MR (2011). Protein-protein interaction networks and subnetworks in the biology of disease. Wiley Interdiscip Rev Syst Biol Med.

[22] Glazko GV, Emmert-Streib F (2009). Unite and conquer: univariate and multivariate approaches for finding differentially expressed gene sets. Bioinformatics.

[23] Khatri P, Sirota M, Butte AJ (2012). Ten years of pathway analysis: current approaches and outstanding challenges. PLoS Comput Biol.

[24] Li Y, Agarwal P (2009). A pathway-based view of human diseases and disease relationships. Plos One.

[25] Xia Y, Yu H, Jansen R, Seringhaus M, Baxter S, Greenbaum D (2004). Analyzing cellular biochemistry in terms of molecular networks. Biochemistry.

[26] Blow JJ, Gillespie PJ (2008). Replication licensing and cancer (mdash) a fatal entanglement?. Nat Rev Cancer.

[27] Halazonetis TD (2010). Genomic instability - an evolving hallmark of cancer. Nat Rev Mol Cell Biol.

[28] Bester A, Roniger M, Oren Y, Im M, Dan S, Chaoat M (2011). Nucleotide deficiency promotes genomic instability in early stages of cancer development. Cell.

[29] Pillaire MJ, Selves J, Gordien K, Gouraud PA, Gentil C, Danjoux M (2009). A ‘DNA replication' signature of progression and negative outcome in colorectal cancer. Oncogene.

[30] Wang ET, Sandberg R, Luo S, Khrebtukova I, Zhang L, Mayr C (2011). Alternative isoform regulation in human tissue transcriptomes. Nature.

[31] Pan Q, Shai O, Lee LJ, Frey BJ, Blencowe BJ (2009). Deep surveying of alternative splicing complexity in the human transcriptome by high-throughput sequencing. Nat Genet.

[32] Tazi J, Bakkour N, Stamm S (2009). Alternative splicing and disease. Biochim Biophys Acta.

[33] Shukla S, Kavak E, Gregory M, Imashimizu M, Shutinoski B, Kashlev M (2011). CTCF-promoted RNA polymerase II pausing links DNA methylation to splicing. Nature.

[34] Savage KI, Gorski JJ, Barros EM, Irwin GW, Manti L, Powell AJ (2014). Identification of a BRCA1-mRNA splicing complex required for efficient DNA repair and maintenance of genomic stability. Mol Cell.

[35] Williamson D, Lu YJ, Fang C, Pritchard-Jones K, Shipley J (2006). Nascent pre-rRNA overexpression correlates with an adverse prognosis in alveolar rhabdomyosarcoma. Genes Chromosomes Cancer.

[36] Bernstein KA, Bleichert F, Bean JM, Cross FR, Baserga SJ (2007). Ribosome biogenesis is sensed at the Start cell cycle checkpoint. Mol Biol Cell.

[37] Urrego D, Tomczak AP, Zahed F, Stuhmer W, Pardo LA (2014). Potassium channels in cell cycle and cell proliferation. Philos Trans R Soc Lond B Biol Sci.

[38] Donati G, Brighenti E, Vici M, Mazzini G, Treré D, Montanaro L (2011). Selective inhibition of rRNA transcription downregulates E2F-1: a new p53-independent mechanism linking cell growth to cell proliferation. J Cell Sci.

